# Bradycardia, Renal Dysfunction, Atrioventricular Nodal Blockade, Shock, and Hyperkalemia (BRASH) Syndrome: A Case Report Highlighting the Importance of Early Recognition and Management

**DOI:** 10.7759/cureus.55892

**Published:** 2024-03-10

**Authors:** Mylena M Lopes Ideta, Franciane P Kühl, Julia Gaio, Rafael M Miyazima

**Affiliations:** 1 Department of Internal Medicine, Federal University of Parana, Curitiba, BRA

**Keywords:** hyperkalemia, cardiogenic shock, bradycardia, acute kidney injury, atrioventricular block

## Abstract

BRASH syndrome, characterized by bradycardia, renal dysfunction, atrioventricular nodal blockade, shock, and hyperkalemia, is a newly defined condition that can lead to significant morbidity and mortality if not promptly recognized and treated. The triggers for this syndrome often include medication interactions, dehydration, and nephrotoxic insults, particularly in older patients with limited renal reserve and cardiovascular disease. In this report, we present the case of an 88-year-old female with multiple comorbidities who exhibited symptoms of prostration, bradycardia, hypotension, and altered mental status, along with laboratory findings (hyperkalemia and renal dysfunction) consistent with BRASH syndrome, triggered by hypovolemia associated with a urinary tract infection. Immediate treatment must focus on correcting hyperkalemia, providing hemodynamic support for bradycardia and hypotension, and administering guided fluid resuscitation. Prompt identification and management of the syndrome can prevent the need for invasive interventions, such as pacemaker insertion and dialysis. Healthcare professionals should be vigilant in considering BRASH syndrome, especially in older patients with cardiac disease, limited renal function, and those on medication regimens that include AV-nodal blocking agents, angiotensin-converting enzyme inhibitors, angiotensin receptor blockers, and potassium-sparing diuretics. This case report emphasizes the importance of clinical suspicion and the initiation of timely treatment to interrupt the cycle of BRASH syndrome and improve patient outcomes.

## Introduction

BRASH (bradycardia, renal dysfunction, atrioventricular nodal blockade, shock, and hyperkalemia) syndrome has been newly defined as atrioventricular nodal blocking and renal dysfunction, associated with hyperkalemia that exacerbates bradycardia and leads to hypoperfusion and shock. The components of this syndrome are interdependent, creating a vicious cycle that can perpetuate itself if not identified [1,2]. BRASH syndrome is underrecognized and can result in high morbidity if not recognized and treated appropriately, leading to cardiovascular collapse and multisystem organ failure [3,4]. Therefore, reporting BRASH syndrome is crucial to improve the ability of healthcare professionals to recognize and appropriately treat this syndrome.

## Case presentation

An 88-year-old female was found in a prostrate position. Upon admission, she was noted to have nausea, vomiting, altered mentation, and bradycardia. She had previously been diagnosed with essential hypertension, type 2 diabetes mellitus, dyslipidemia, hypothyroidism, chronic renal failure (Kidney Disease Improving Global Outcomes G3a classification), ischemic stroke, and osteoporosis. Her outpatient medication regimen included insulin (12 IU daily), levothyroxine (100 µg daily), losartan (50 mg twice daily), metoprolol (50 mg daily), amiodarone (200 mg daily), simvastatin (40 mg daily), metformin (500 mg twice daily), and omeprazole (20 mg daily).

Initial vital signs revealed a temperature of 35.6°C, blood pressure of 90/44 mmHg with a mean arterial pressure of 59 mmHg, heart rate of 45 beats per minute, oxygen saturation of 92% requiring supplementary nasal oxygen, and a regular respiratory rate. On physical examination, she exhibited dehydrated skin, isochoric and photoreactive pupils, and confusion (with a Glasgow Coma Scale score of 14), but no focal neurological deficits. Her extremities were cold, with the presence of livedo reticularis (Mottling score of 3), and no edema. Blood tests revealed hyperlactatemia, metabolic acidosis, hyperkalemia, renal dysfunction, and microcytic anemia (Table 1).

**Table 1:** Laboratory values from baseline, admission, and hospital discharge. Hb = hemoglobin; WBC = white blood cell count; TSH = thyroid-stimulating hormone

	Baseline (two weeks before admission)	Admission	Discharge	Reference value
Creatinine (mg/dL)	1.05	1.88	1.0	0.57–1.11 mg/dL
Potassium (mEq/L)	4.8	7.1	4.6	3.5–5.1 mEq/L
Sodium(mEq/L)	137	131	137	136–145 mEq/L
WBC (/μL)	5,920	14,500	3,550	3,800–11,000/μL
Hb (g/dL)	8.7	8.4	10.1	12.5–15.7 g/dL
C-reactive protein (mg/dL)	-	0.26	0.42	<0.5 mg/dL
Bicarbonate (mmol/L)	-	12	26	23–29 mmol/L
Lactate (mmol/L)	-	6.8	-	0.36–1.39 mmol/L
TSH (μUI/mL)		5.0		
Free thyroxine (ng/dL)		1.6		

**Figure 1:**
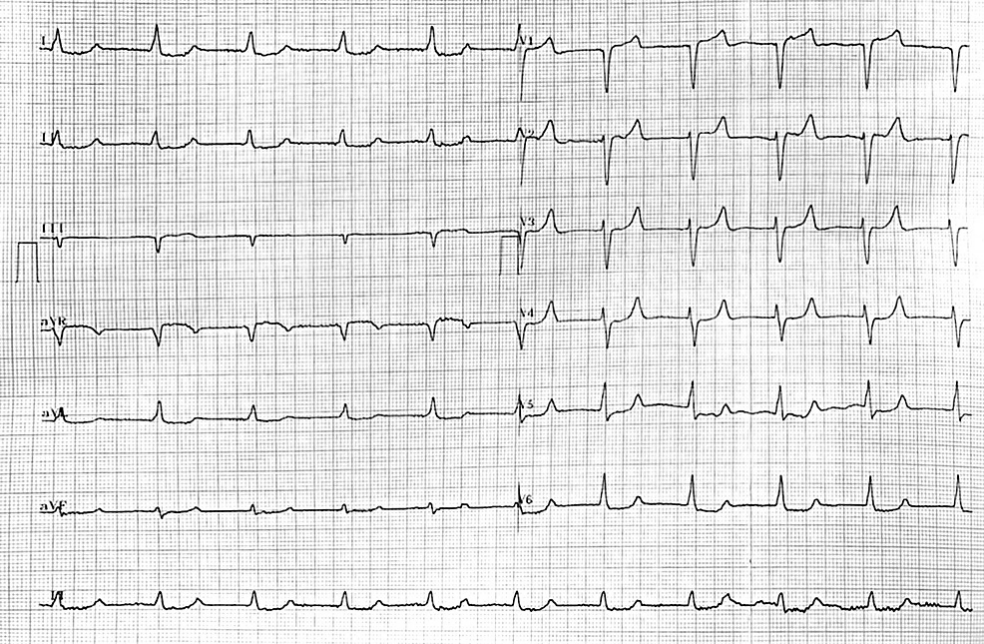
Electrocardiogram on admission.

The initial electrocardiogram (EKG) demonstrated the presence of sinus bradycardia, which we decided to manage with atropine, with unsuccessful results. The patient continued presenting bradycardia and hypotension, leading to the initiation of norepinephrine. Subsequently, a junctional pattern developed, accompanied by peaked T waves, mainly from V2 to V4, depression of the ST segment from V4 to V6, and diffuse nonspecific ST-segment abnormalities. QRS prolongation and ST shortening were not present as in classic cases of significant hyperkalemia (Figure 1). Myocardial injury was investigated with troponin levels which showed no elevation (14.4 pg/mL; 17.2 pg/mL). Point-of-care ultrasound revealed a bilateral A-line pattern with lung sliding, cardiac output was estimated at 1.6 L/minute, and the collapsibility index of the inferior vena cava was 24%. Given the suspicion of a cardiogenic shock with a component of hypovolemic shock, dobutamine and intravenous fluid were administered. The patient was admitted to the intensive care unit (ICU) two hours after arrival. Urgent echocardiography was performed in the ICU and was compatible with normal biventricular systolic function with a left ventricular ejection fraction of 75% during dobutamine administration.

The diagnosis of BRASH syndrome was established due to the presence of bradycardia, renal dysfunction, atrioventricular disturbances, shock, and hyperkalemia. Consequently, a conservative approach was adopted, focusing on the clinical management of hyperkalemia (with intravenous calcium and insulin plus glucose), intravenous fluid therapy, and hemodynamic support using vasoactive agents. The patient’s clinical state declined over the following hours, developing seizures and a critical worsening of her neurological state, associated with pyuria and fever. Neutrophil left shift was observed in the patient’s examinations after admission. This led to advanced airway management and ceftriaxone administration because of urinary tract infection suspicion. Hemocultures and urine cultures were collected. *Klebsiella variicola* was identified in the urine culture, and the antibiotic regimen was maintained to complete treatment.

The patient was successfully weaned off vasoactive agents and mechanical ventilation, showing favorable clinical and laboratory improvement. After 15 days in the ICU, the patient was discharged to the hospital ward rehabilitation and to prepare for discharge with planned outpatient cardiology follow-up. At the time of discharge, the EKG showed a normal sinus rhythm, serum potassium was measured at 4.6 mEq/L, and creatinine levels were noted to be 1.0 mg/dL.

## Discussion

Our patient, an 88-year-old female, had a history of using beta-blockers (metoprolol) and angiotensin receptor blockers (losartan) and presented with bradycardia and dehydration. Laboratory findings revealed hyperkalemia and acute kidney injury. The combination of bradycardia, renal dysfunction, atrioventricular disturbances, shock, and hyperkalemia led to the diagnosis of BRASH syndrome. We considered that both hypovolemia and urinary tract infection were triggers for the deterioration of renal function and, therefore, starting the vicious cycle. Treatment focused on supporting the patient’s clinical conditions, volume resuscitation, and antibiotic therapy. The inpatient mortality associated with BRASH syndrome was 5.7% based on published case reports [5]. By recognizing the syndrome and providing appropriate supportive care, we were able to avoid invasive procedures such as permanent pacemaker insertion and dialysis and potentially fatal outcomes for this patient.

BRASH syndrome was first described by Josh Farkas in 2016 [1]. Hyperkalemia and atrioventricular-blocking medications exert a synergistic effect on hemodynamics, resulting in decreased cardiac output, shock, and impaired renal perfusion, which further elevate potassium levels and lead to the accumulation of renally cleared atrioventricular-blocking medications, perpetuating the vicious cycle. Nonselective beta-blockers also present hyperkalemia as a collateral effect. Angiotensin-converting enzyme inhibitors and angiotensin receptor blockers act as contributors to kidney dysfunction and hyperkalemia in those patients. Specifically, in this case, losartan was associated with elevated serum potassium levels and deteriorating renal function.

This syndrome is usually present in older patients with cardiac disease and limited renal reserve [5]. The potential triggers reported are usually mild clinical events, such as medication up-titration, exposure to nephrotoxins, use of potassium-sparing diuretics, and dehydration. Certain medications can induce this syndrome, with examples including angiotensin-converting enzyme inhibitors, angiotensin receptor blockers, digitalis, beta-blockers, and non-dihydropyridine calcium channel blockers [2,6,7]. In some cases, medications such as amiodarone and metoprolol may exacerbate bradycardia. A systematic review of case reports described that 75.7% of the patients presented signs of central nervous system hypoperfusion as fatigue, headache, somnolence, encephalopathy, and syncope. Other symptoms included abdominal pain, vomiting, diarrhea, dyspnea, and chest pain. The mean serum potassium concentration was 5.2 mEq/L. The most common EKG finding was a junctional escape rhythm. Our patient findings were compatible with the data available in this review [5].

Hyperkalemia in BRASH syndrome cases is an important contributor to cardiac conduction abnormalities, which differentiates it from “simple” nodal atrioventricular blockade toxicity [5,8]. The treatment is based on the pathophysiology of the syndrome and must include prompt correction of hyperkalemia, based on intravenous calcium for myocardial membrane stabilization, intravenous rapid-acting insulin, and sodium bicarbonate infusion. Dialysis may be required as a definitive measure for controlling hyperkalemia if the patient is refractory to clinical management [2]. Management of circulatory shock and supportive cardiac care should be done for all patients. Most patients respond to vasopressors and inotropic therapy, although approximately one-third of patients may need temporary transcutaneous or transvenous cardiac pacing [5]. Fluid resuscitation may be performed guided by the patient’s hemodynamic parameters [2,6,9].

## Conclusions

BRASH syndrome components are interdependent, creating a vicious cycle that can perpetuate itself if not identified. Therefore, early recognition of BRASH syndrome enables the prompt correction of its components, which improves the prognosis and decreases the likelihood of requiring invasive interventions. Our patient presented with hypovolemic shock associated with cardiogenic shock as a complication of the syndrome, making immediate interventions such as volume resuscitation, vasopressor use, and ICU monitoring necessary.

This case report underscores the importance of considering BRASH syndrome in patients presenting with characteristic symptoms and laboratory abnormalities. Such consideration allows for appropriate management aimed at breaking the cycle and preventing complications. Healthcare professionals should be vigilant in considering BRASH syndrome, especially in older patients with cardiac disease, limited renal function, and medication regimens that include atrioventricular nodal-blocking agents, angiotensin-converting enzyme inhibitors, angiotensin receptor blockers, and potassium-sparing diuretics. Further research is imperative to enhance the comprehension and recognition of this syndrome in clinical practice, ultimately leading to improved outcomes.
